# The burden and impact of COVID-19 among newborns in African countries: a study protocol for a systematic review and meta-analysis

**DOI:** 10.1093/inthealth/ihad112

**Published:** 2023-12-11

**Authors:** Teklehaimanot Gereziher Haile, Tamirat Getachew, Assefa Iyasu Negash

**Affiliations:** Department of Maternity and Neonatal Nursing, School of Nursing, College of Health Sciences and Comprehensive Specialized Hospital, Aksum University, Aksum, Tigray, Ethiopia; School of Nursing and Midwifery, College of Health and Medical Sciences, Haramaya University, Harar, Ethiopia; Department of Adult Health Nursing, School of Nursing, College of Health Sciences and Comprehensive Specialized Hospital, Aksum University, Aksum, Tigray, Ethiopia

**Keywords:** Africa, burden, COVID-19, impact, newborn

## Abstract

The WHO, on 30 January 2020, declared the Chinese outbreak of coronavirus disease 2019 (COVID-19) a global community health emergency that poses a serious threat to vulnerable healthcare systems. This review protocol will be conducted to systematically review and to perform a meta-analysis on the impact of COVID-19 among newborns in Africa. All observational studies on the impact of COVID-19 among newborns in Africa will be included. A standard quest strategy to retrieve studies was conducted on several databases (Google Scholar, PubMed/MEDLINE, EMBASE, HINARI, Cochrane Library, WHO COVID-19 database, Africa Wide Knowledge and Web of Science). Two independent authors were tasked to extract key data and to assess the risk of bias. To assess possible publication bias, funnel plot test and Egger's test methods will be used. The description will be used to show the COVID-19 distribution data by interest variables such as residence, setting and person-level characteristics. The findings of this review will notify healthcare professionals about the burden and impact of COVID-19 and provide evidence to bring about the requisite improvements in clinical practice.

## Introduction

Severe acute respiratory syndrome coronavirus-2 (SARS-CoV-2), the pathogen responsible for the coronavirus disease 2019 (COVID-19) outbreak, originated in late December 2019 in Wuhan City, China, and is highly infectious with rapid human-to-human spread.^1,2^ The WHO, on 30 January 2020, proclaimed the Chinese outbreak of COVID-19 as a global public health emergency that poses a serious threat to vulnerable health systems.^3^ The WHO, in February 2020, labelled the disease ‘COVID-19’, and the International Committee on Virus Classification named the virus ‘severe acute respiratory syndrome coronavirus-2’ (or SARS-CoV-2).^4,5^ On 11 March 2020, the WHO declared COVID-19 a pandemic.^6^

COVID-19 is documented to be transmitted from both symptomatic and asymptomatic patients by direct touch, aerosol droplets, fecal-oral and intermediate fomites.^7,8^ The symptoms of the disease include fever, dry cough, difficulty breathing and diarrhoea in 20–25% of patients without upper respiratory symptoms like sneezing or sore throat.^2^ Some develop multiple fatal complications such as septic shock, life-threatening pneumonia and organ failure.^9^

The incubation period is typically 1–14 d, with a medium length of 3–7 d and up to 24 d in a small minority of cases.^10,11^ To date, COVID-19 fatality rates range from 1% to >7%, and the key cause remains respiratory failure; however, the full course of the disease is not yet understood.^12^

According to WHO situation reports, on 6 August 2020, the number of reported cases of COVID-19 was 19 117 400 cases worldwide, with 713 911 deaths in 215 countries. The USA has the largest number of confirmed cases with 4 996 426 cases and 162 017 deaths, followed by Brazil with 2 873 304 cases and 97 692 deaths.^6^ By 6 August 2020, COVID-19 had affected approximately 57 African countries, with 1 002 181 confirmed cases, 21 771 deaths and 680 960 recoveries. South Africa is the most affected African country, with a total number of reported cases of 529 877 and 9298 deaths.^6^

According to a study conducted among university students in Saudi Arabia, the COVID-19 epidemic has significantly increased the symptoms of depression in students, particularly in female students.^13^ According to a Chinese study, public health expenditure and policy were crucial to the management and containment of the COVID-19 pandemic in China.^14^

Based on a Russian study, using such sanitary guidelines can help prevent public health hazards in both common and very difficult self-isolation scenarios. The primary nutritional deficits (malnutrition), hypoxia, work/rest imbalance and sedentary lifestyle are the sanitary hygiene risk factors of self-isolation.^15^ According to an Indian study, despite many war-footing measures to combat the virus, COVID-19 has added an extra burden to the already overstretched healthcare infrastructure. As a result, the number of infected cases and deaths has increased dramatically, placing India in second place out of the 10 nations most affected by COVID-19.^16^

With respect to children, on 8 February 2020, a 13-mo-old child was registered as being the first serious case. In addition, a 17-d-old newborn was first identified on 5 February 2020 as a neonatal infection with positive SARS-CoV-2 with pharyngeal swabs and anal swabs.^17,18^

The rapid development of the COVID-19 pandemic has created tremendous obstacles for both the public and healthcare professionals worldwide. Considering newborns and children are vulnerable to infectious diseases, a lot of attention is paid to the prevalence of the disease among them. In dealing with the cases in neonates and infants, as well as a stable paediatric age group, the strategy shapes comprehensive strategies to counter the novel coronavirus disease. There have been different reports about COVID-19.^19–21^ However, there are no pooled results of the burden and impact of COVID-19 among newborns in Africa. This review protocol will be applied to systematically review and conduct a meta-analysis of the impact of COVID-19 among newborns in Africa.

## Methodology

### Registration of protocol

This study protocol was registered in the PROSPERO Registry of Systematic reviews in August 2020 and was accepted with the registration number CRD42020202666 (https://www.crd.york.ac.uk/PROSPERO) and reported according to Preferred Reporting Items for Systematic reviews and Meta-Analysis protocol (PRISMA-P) guidelines^22^ (Table 1).

**Table 1.** PRISMA-P 2015 checklist: recommended items to address in a systematic review protocol

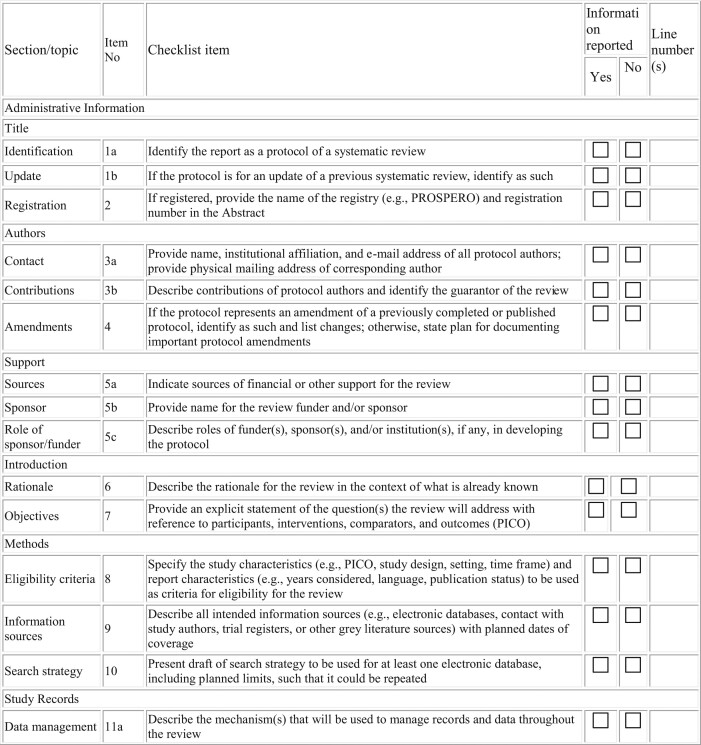
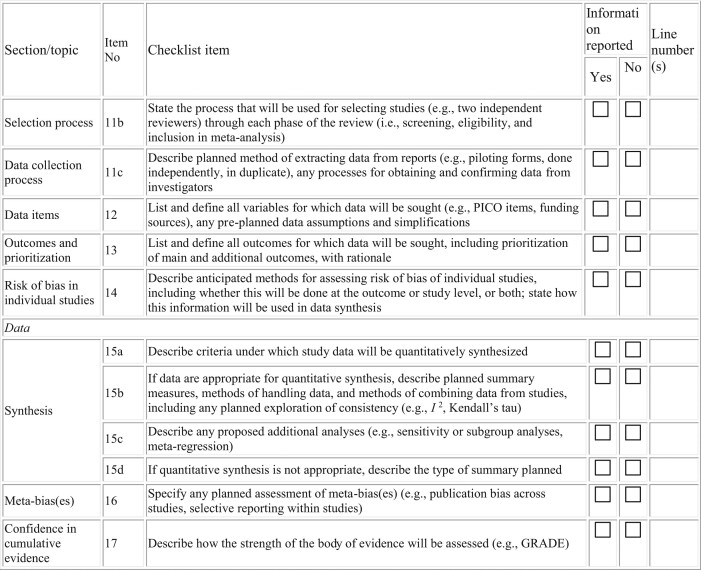

### Criteria for eligibility

**Study type:** All observational studies including cohort, case-control, cross-sectional studies and baseline results from randomised controlled trials will be carried out in this protocol.

**Participants:** All newborns who are of African residence and have laboratory-confirmed and/or clinically diagnosed COVID-19.

**Intervention(s), exposure(s):** Newborns with an infection of COVID-19. Therefore, we need to evaluate disease severity, mortality, burden and impact.

**Outcomes:** Mortality and clinical impacts of COVID-19 among newborns (clinical characteristics, infection rate, prevalence rate, burden and impact of COVID-19).

**Setting:** Institutional-based studies.

**Method of diagnosis**: Subgroup analysis will be carried out with no restriction on diagnostic instruments. Interim guidance from the WHO and/or any diagnostic criteria proposed by the WHO shall be considered: ‘WHO interim guidance for laboratory biosafety related to 2019-nCoV’.^23,24^

**Exclusion criteria:** Studies that did not clarify the requirements for the burden and impact of COVID-19 level, studies that have not been conducted in humans, qualitative studies, as well as studies that lack valid data required to determine the outcome, will not be included. Additionally, studies such as experimental studies, commentaries, editorials, letters, case reports or case series will be excluded from this review.

### Source of data and search strategy

Online databases such as Google Scholar, PubMed/MEDLINE, EMBASE, HINARI, Cochrane Library, WHO COVID-19 database, Africa Wide Knowledge and Web of Science were used as search techniques for the impact of COVID-19 (Table 2) using the following keywords as search terms: burden, impact, coronavirus diseases (COVID-19), Africa, infant and newborn. Additional search terms such as ‘Wuhan coronavirus’ OR ‘COVID-19’ OR ‘novel coronavirus’ OR ‘2019-nCoV’ OR ‘Coronavirus outbreak’ OR ‘SARS-CoV-2’ OR ‘SARS2’ OR ‘Severe acute respiratory syndrome coronavirus 2’ OR ‘Burden’ OR ‘Impact’ will be used. Other terms that will be searched are ‘prevalence’ OR ‘mortality’ OR ‘occurrence’ OR ‘impact of COVID-19 in newborns’.

**Table 2.** Search strategy

S. N	Online searching databases	No. of studies found	No. of studies involved	No. of studies omitted	Reason for omission
1	Google Scholar	N	N	N	
2	PubMed/MEDLINE	N	N	N	
3	EMBASE	N	N	N	
4	HINARI	N	N	N	
5	Cochrane Library	N	N	N	
6	WHO COVID-19 database	N	N	N	
7	Africa Wide Knowledge	N	N	N	
8	Web of Science	N	N	N	
9	Unpublished thesis, manuscript, and report from WHO and CDC	N	N	N	

The search technique was carried out by two separate scholars. Article-searching procedure will be established using the captions of the scientific subject being used (MESH) and the operative BOOLEAN (AND/OR). This research review will be drawn up with the ‘COVID-19 Open Study Dataset’ metadata (updated August 2020).

### Selection and data collection process

Data will be extracted using a standardised method of data extraction. Two assessors (TGH and TG) will extract the data autonomously using a predefined and standardised method of data extraction from the included articles. Full texts with titles, abstracts and those with ambiguity will be collected to assess whether they are included or not in this review. Cohen's λ statistics calculation will be used to check the agreement between the reviewers of this study. Resolution of disagreements will be carried out through discussion, arbitration and mediation by a third reviewer (AIN). After the exclusion of studies, the reasons will be noted.

To ensure study suitability, for the missing data, authors will be contacted to provide additional details. To demand further information before excluding any study, three emails will be sent to the corresponding author (if necessary). We will consider studies such as the most recent and detailed with the largest sample size that look at >1 published study. For surveys that appear in one study with multiple surveys conducted at different times, we should treat each survey as a separate study.

Information such as the first author, country, publishing month, signs and symptoms, complications, diagnostic criteria, comorbidity, COVID-19 prevalence or incidence and characteristics of the study will be included during data extraction.

### Assessment of quality and risk bias

A tool developed by Hoy et al. for prevalence studies will be used to assess the likelihood of bias and the quality of the studies included in this review.^25^ The tool contains 11 items; items 1–4 assess the external validity, 5–10 assess the internal validity and item 11 offers a description of the overall risk by the reviewer based on the responses of the above 10 items, which are rated 1 if yes and 0 if no. Studies are graded as low (<3), moderate risk^4–6^ or high^7–9^ risk of bias.

Two reviewers will conduct this exercise, and disputes will be resolved through discussion, and, where possible, through arbitration involving a third author. In addition, adequate sampling methods, consistent methods and procedures for collecting data, recorded methods of quality control and representative sample size, will be considered as indicators of study quality. High quality studies will be those studies that reveal all the points mentioned above. We will use the SPIRIT 2013 checklist^26^ for randomised clinical trials. This is further explained in additional file 1 on the SPIRIT checklist.

### Management of data

A framework was developed a priori to guide the screening and selection process, based on the inclusion and exclusion criteria. The tool will be piloted and revised before data extraction begins. First, to delete duplicates, the search results were uploaded to EndNote software. The remaining articles will be put on Rayyan, a smartphone and web-based software system that facilitates collaboration between reviewers involved in the screening and selection of studies to be included in the review.^27^

### Data items

Authors, month, country and/or region, sample size, publication type, research field, research characteristics (study design, response rate, average or median age or age range) will be included in data extraction.

### Outcomes and prioritisation

The primary outcome of this study is to assess the burden and impact of COVID-19 among newborns in African countries. This includes examining the incidence and prevalence of COVID-19 infection among newborns, as well as the associated morbidity and mortality rates. Secondary outcomes of this study protocol focus on investigating the rates of maternal–neonatal transmission of COVID-19, clinical presentation of COVID-19 in newborns, as well as complications and comorbidities associated with COVID-19 infection among newborns in African countries. It includes assessing the incidence of respiratory distress syndrome, pneumonia, sepsis and other related complications.

### Data analysis, synthesis and result presentation

In the data analysis process, version 3.5.3 of R software and version 1.2.5003 of R studio will be used. Where appropriate, data will be summarised using range, mean±SD and frequency (%). All analyses will be performed using the ‘meta-prop’ routines of Windows version R 3.5.3.^28^ Results will be reported as proportion with the corresponding 95% CI. Forest plots will be drawn to represent the combined burden and impact of COVID-19 and the extent of statistical heterogeneity among studies.

The statistical heterogeneity will be evaluated using the χ^2^ test and quantified using calculation of the *I*^2^ statistics with values of 25%, 50% and 75% being representative of low, medium and high heterogeneity, respectively.^29^ If there will be heterogeneity between studies. We will use a random effects model of meta-analysis^30^ to estimate the combined impact of COVID-19 in African countries. To assess possible publication bias, we will use the funnel plot test and Egger’s test.^31^ p<0.10 in Egger's test is considered statistically significant, indicating the presence of bias in the study.

### Data synthesis

The study-specific burden and impact of COVID-19 among newborns will be recalculated using crude numerators and denominators from the individual studies. A meta-analysis will be performed on variables that are similar across the included studies. If heterogeneity is present among the studies, the random effect model will be used to determine the pooled impact of COVID-19 in Africa. African geographical regions, diagnostic methods and the ethnic background where the study was conducted will be summarised by a subgroup analysis.

## Discussion

**Figure 1.**
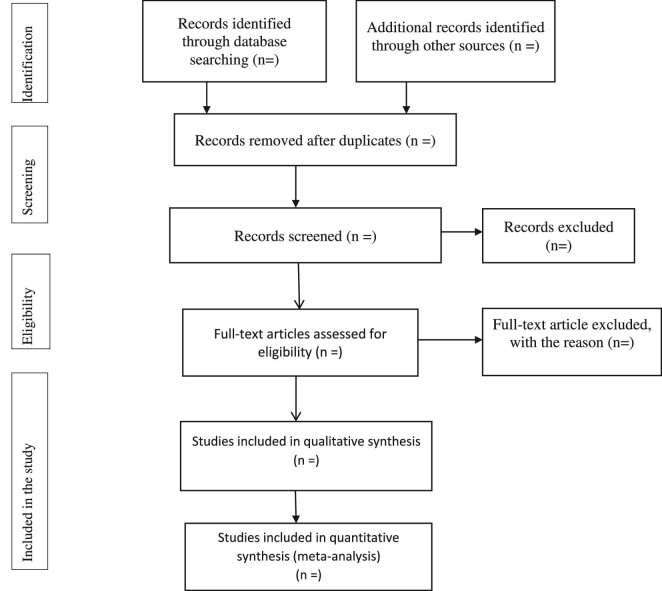
Selection of articles for a systematic review and meta-analysis on the burden and impact of COVID-19 among newborns in African countries.

This study protocol will be published in accordance with PRISMA-P guidelines, and the PRISMA flow diagram will be used to document the different phases of the review process^22^ (Figure 1).

The description will be used to show the COVID-19 distribution data by interest variables such as residence, setting and person-level characteristics. A funnel plot will be used to illustrate the publication bias of the studies. A forest plot will be used for the included studies to predict the burden and impact of COVID-19 among newborns in Africa. The findings of this review will notify healthcare professionals about the burden and impact of COVID-19 and provide evidence to bring about the requisite improvements in clinical practice. Conferences, peer-reviewed articles and social media sites will share conclusions from this study.

### Conclusion

This study protocol is expected to show the burden and impact of COVID-19 among newborns in African countries.

## Data Availability

The dataset and/or analysis used during the study will be provided in the manuscript and can be provided upon request to the corresponding author.
